# Glucose Metabolic Reprogramming of ER^+^ Breast Cancer in Acquired Resistance to the CDK4/6 Inhibitor Palbociclib

**DOI:** 10.3390/cells9030668

**Published:** 2020-03-10

**Authors:** Nicla Lorito, Marina Bacci, Alfredo Smiriglia, Michele Mannelli, Matteo Parri, Giuseppina Comito, Luigi Ippolito, Elisa Giannoni, Martina Bonechi, Matteo Benelli, Ilenia Migliaccio, Luca Malorni, Paola Chiarugi, Andrea Morandi

**Affiliations:** 1 Department of Experimental and Clinical Biomedical Sciences, University of Florence, Viale Morgagni 50, I-50134 Florence, Italy; nicla.lorito@gmail.com (N.L.); marina.bacci@unifi.it (M.B.); alfredo.smiriglia@stud.unifi.it (A.S.); michele.mannelli@student.unisi.it (M.M.); matteo.parri@unifi.it (M.P.); giuseppina.comito@unifi.it (G.C.); luigi.ippolito@unifi.it (L.I.); elisa.giannoni@unifi.it (E.G.); paola.chiarugi@unifi.it (P.C.); 2 Translational Research Unit, Azienda USL Toscana Centro, Hospital of Prato, Via Suor Niccolina Infermiera 20, I-59100 Prato, Italy; martina.bonechi@uslcentro.toscana.it (M.B.); ilenia.migliaccio@uslcentro.toscana.it (I.M.); luca.malorni@uslcentro.toscana.it (L.M.); 3 Bioinformatics Unit, Azienda USL Toscana Centro, Hospital of Prato, Via Suor Niccolina Infermiera 20, I-59100 Prato, Italy; matteo.benelli@uslcentro.toscana.it; 4 “Sandro Pitigliani” Oncology Department, Azienda USL Toscana Centro, Hospital of Prato, Via Suor Niccolina Infermiera 20, I-59100 Prato, Italy

**Keywords:** palbociclib, metabolic reprogramming, breast cancer, Warburg metabolism, resistance

## Abstract

The majority of breast cancers express the estrogen receptor (ER) and are dependent on estrogen for their growth and survival. Endocrine therapy (ET) is the standard of care for these tumors. However, a superior outcome is achieved in a subset of ER positive (ER^+^)/human epidermal growth factor receptor 2 negative (HER2^−^) metastatic breast cancer patients when ET is administrated in combination with a cyclin-dependent kinases 4 and 6 (CDK4/6) inhibitor, such as palbociclib. Moreover, CDK4/6 inhibitors are currently being tested in ER^+^/HER2^+^ breast cancer and reported encouraging results. Despite the clinical advances of a combinatorial therapy using ET plus CDK4/6 inhibitors, potential limitations (i.e., resistance) could emerge and the metabolic adaptations underlying such resistance warrant further elucidation. Here we investigate the glucose-dependent catabolism in a series of isogenic ER^+^ breast cancer cell lines sensitive to palbociclib and in their derivatives with acquired resistance to the drug. Importantly, ER^+^/HER2^−^ and ER^+^/HER2^+^ cell lines show a different degree of glucose dependency. While ER^+^/HER2^−^ breast cancer cells are characterized by enhanced aerobic glycolysis at the time of palbociclib sensitivity, ER^+^/HER2^+^ cells enhance their glycolytic catabolism at resistance. This metabolic phenotype was shown to have prognostic value and was targeted with multiple approaches offering a series of potential scenarios that could be of clinical relevance.

## 1. Introduction

The majority of breast tumors are positive for estrogen receptor alpha (ER) and negative for human epidermal growth factor receptor 2 (HER2) and are, therefore, dependent on estrogen (E2) for their growth. Different endocrine therapies (ET) have been developed to target the ER pathway and have been used effectively in the clinic in the last decades. Recently, clinical trials have shown superior outcomes in ER^+^/HER2^−^ patients receiving a cyclin-dependent kinases 4 and 6 (CDK4/6) inhibitors in combination with standard ET [1,2,3,4]. This led to regulatory approval for the use of CDK4/6 inhibitors in the clinic for patients with ER^+^/HER2^−^ metastatic breast cancers both as first- and second-line therapy [5] contributing to their widespread use within this indication. It is foreseen that the use of these compounds will increase in the future, potentially extending their application to the whole cohort of ER^+^ patients.

Palbociclib (Ibrance, PD-0332991), the first CDK4/6 inhibitor synthesized and tested in clinical trials, is currently indicated in combination with ET both as first- and second-line therapeutic option in patients with ER^+^/HER2^−^ breast cancer [5] and is under investigation for the treatment of patients with ER^+^/HER2^+^ disease. However, despite the promising clinical advances with CDK4/6 inhibitors, 15% of the ER^+^/HER2^−^ breast cancer patients show de novo or early CDK4/6 inhibitors resistance and 50% develop clinical resistance with progression within 25 months [6]. Understanding the mechanisms involved in CDK4/6 inhibitors’ resistance is, therefore, an important biological and clinical need.

It has been previously reported that CDK4/6 inhibitors’ resistance is characterized by deregulated molecular mechanisms that could occur on cell-cycle related hubs, e.g., loss of Rb, amplification of p16, CDK2, 4, 6, CCNE1, 2, and overexpression of CDK7, MDM2, WEE1. These mechanisms have been extensively reviewed in [7,8]. Moreover, emerging data in preclinical models have indicated that metabolic deregulation could guide palbociclib response [9] suggesting that metabolic reprogramming could be involved also in the establishment of the resistant phenotype.

Importantly, metabolic deregulation occurs in tumorigenesis and targeting bioenergetic alterations sensitizes cancer cells to chemo- and biological therapies. Since CDK4 and CDK6 control key metabolic regulators in physiological and pathological conditions and manipulating metabolic pathways in addition to CDK4/6 inhibition is beneficial in cancer [9,10,11,12], we investigated the metabolic reprogramming that occurs in a panel of isogenic ER+ breast cancer cell lines in response and adaptation to palbociclib.

The current study highlights the metabolic changes occurring in glucose exploitation in ER^+^ breast cancer cells that are exposed (+PD) or have acquired resistance to palbociclib (PDR). Crucially, we demonstrated that ER^+^/HER2^+^ and ER^+^/HER2^−^ breast cancer cells rely on aerobic glycolysis differently when mimicking a sensitive or a resistant clinical scenario, hence targeting glucose metabolism can re-sensitize ER^+^/HER2^+^ PDR cells to palbociclib and significantly enhance its anti-tumoral effects in ER^+^/HER2^−^ sensitive cells.

## 2. Materials and Methods

### 2.1. Cell Lines and Reagents

T47D and ZR75-1 (ER^+^/HER2^−^) human breast cancer cell lines were obtained from Dr Livia Malorni, CNR Avellino, Italy; MCF7 (ER^+^/HER2^−^) human breast cancer cells were purchased from ATCC; ER^+^/HER2^+^ MDA-MB-361 cells were purchased from Sigma-Aldrich and BT474 cells from Interlab Cell Line Collection, Genova, Italy. In January 2016, all cell lines and their PDR derivatives have been authenticated by short tandem repeat DNA profiling analysis. Cell lines were maintained in Dulbecco’s Modified Eagle Medium (DMEM, Euroclone, Pero, Italy) supplemented with 10% fetal bovine serum (FBS, Euroclone), 2 mM glutamine (Sigma, Merck, Darmstadt, Germany) and 1% penicillin/streptomycin (Sigma). The palbociclib-resistant derivatives (PDR cells) were cultured in the same condition in the presence of 1 µM palbociclib (Pfizer, Pfizer Italia, Latina, Italy), as previously described [13]. Cells were amplified, stocked, frequently subjected to mycoplasma testing and once thawed were kept in culture for a maximum of 20 passages. To evaluate cell viability under glucose deprivation or galactose treatment, the cells were cultured in deprived-DMEM 1X (Gibco, Thermo Fisher Scientific, Monza, Italy), supplemented with or without 25 mM glucose (Sigma) in the presence of 10% FBS, 2mM glutamine and 110 mg/L pyruvate (Sigma). 2-deoxy-glucose (2-DG, Sigma) and galactose (Sigma) were used at the concentration of 5 mM and 25 mM, respectively.

### 2.2. Immunoblotting

Breast cancer cells were washed with phosphate-buffered saline (PBS) and lysed in ice using Laemmli Sample Buffer 1X (Biorad, Milano, Italy) supplemented with protease inhibitors (Sigma) and protein concentrations were measured by BCA method. 20–40 µg of total proteins were loaded in precast sodium dodecyl sulfate–polyacrylamide gel electrophoresis (SDS-PAGE) gels (Biorad) and then transferred onto nitrocellulose membrane by Trans-Blot Turbo Transfer Pack (Biorad). The immunoblots were incubated in non-fat dry milk 2%, Tween-20 0.05% in PBS at room temperature for 1 h and then probed with primary and appropriate secondary antibodies. The antibodies used in this work were GLUT1 and HKII (#12939 and #2867, dilution 1:1000, Cell Signaling Technology, Leiden, The Netherlands), MCT1 and MCT4 (sc-365501 and sc-50329, dilution 1:500, Santa Cruz Biotechnology, Heidelberg, Germany) and Tubulin (T5168, dilution 1: 15000, Sigma).

### 2.3. Quantitative Real-Time Polymerase Chain Reaction (qRT-PCR)

Total RNA was extracted using RNeasy (RNeasy plus kit, Qiagen, Milano, Italy), quantified using Nanodrop 1000 (Thermo Scientific) and 500 ng were reverse transcribed, prior to gDNA removal, to obtained cDNA using the QuantiTect cDNA reverse transcription kit (Qiagen). Quantitative real-time polymerase chain reaction (qRT-PCR) was performed using the CFX96 Touch Real-Time PCR Detection System (Biorad) using Taqman gene expression assay (Applied Biosystem, Thermo Fisher Scientific). The probes used are HKII (Hs0060686_m1) and GLUT1 (Hs00892681_m1). Data were normalized on Glyceraldehyde 3-Phosphate Dehydrogenase GAPDH (Hs02786624_g1) and TATA-Box Binding Protein TBP (Hs00427620_m1). The relative quantity was determined using ΔΔCt by the CFX Maestro software (BioRad).

### 2.4. Cell Viability Assays

Parental and PDR cells were seeded into 12- or 24-well plates and subjected to the experimental procedures such as RNA interfering or glycolysis impairment as described in the Figures and relative legends. 3 days post-procedure (i.e., RNAi transfection, drug administration or culture conditions changes), plates were stained with crystal violet (triphenylmethane dye 4-[(4-dimethylaminophenyl)-phenyl-methyl]-*N*,*N*-dimethyl-alanine, Sigma), dried overnight and the crystal violet within the adherent cells was solubilized using 500 μL/well of 2% SDS. The absorbance was evaluated at 595 nm using a microplate reader. Alternatively, cell viability was also assessed by MTT (3-[4,5-dimethylthiazol-2-yl]-2,5-diphenyl tetrazolium bromide, Sigma) assay. Living cells were stained with 10 mg/mL MTT dissolved in full medium and incubated at 37 °C in a humidified atmosphere incubator containing 5% CO_2_ for 3 h. The incorporated amount of MTT was resuspended in dimethyl sulfoxide DMSO and quantified measuring absorbance at 550 nm using a microplate reader.

### 2.5. RNAi Transfection

PDR cells were seeded into 6-well plates (5 × 10^5^ per well) to have a 70% of confluence the day after when cells were transfected with either a 50 nmol/L siRNA targeting HKII (Hs01_00080105, Sigma) or a negative control (SIC001, Sigma) using Lipofectamine RNAiMAX Reagent (Life Technologies, Thermo Fisher Scientific) and Opti-MEM (Gibco) accordingly to the manufacturer’s instructions. The functional analyses described were performed 3 days after transfection.

### 2.6. Radiolabeled Glucose Uptake Assay

Parental and PDR cells were seeded into a 6-well plate in basal culture conditions. Glucose uptake was evaluated incubating the cells with a buffered solution containing 0.2 µCi/mL [^3^*H*]-2-deoxyglucose (Perkin Elmer) for 15 min at 37 °C. Cells were subsequently washed with cold PBS and lysed with 0.1 M NaOH. Incorporated radioactive glucose derived signal was measured by liquid scintillation counting and normalized on protein content.

### 2.7. Gas Chromatography–Mass Spectrometry (GC–MS)

Gas chromatography–mass spectrometry (GC–MS) analysis was previously described [14]. Briefly, for SCAN mode, 1 × 10^6^ cells were collected and subjected to extraction using a mixture of CHCl_3_:MeOH:H_2_O (1:1:1), before quenching with ice-cold MeOH:H_2_O (1:1) containing norvaline (Sigma), used as internal standard. CHCl_3_ was then added, and the samples were vortexed at 4 °C for 30 min, centrifuged at 3000× *g* for 10 min, and the aqueous phase was collected and allowed to evaporate at room temperature. Dried polar metabolites were dissolved in 60 μL of 2% methoxyamine hydrochloride (Sigma) in pyridine (Thermo Fisher Scientific), and held at 30 °C for 2 h. After dissolution and reaction, 90 μL *N*-Trimethylsilyl-*N*-methyl trifluoroacetamide (MSTFA) + 1% trimethylchlorosilane TMCS (Sigma) were added and samples were incubated at 37 °C for 60 min. Gas chromatographic runs were performed with helium as carrier gas at 0.6 mL/min. The split inlet temperature was set to 250 °C and the injection volume of 1 μL. A split ratio of 1:10 was used. The GC oven temperature ramp was from 60 to 325 °C at 10 °C/min. The data acquisition rate was 10 Hz. For the Quadrupole, an Electron Ionization (EI) source (70 eV) was used, and full-scan spectra (mass range from 50 to 600) were recorded in the positive ion mode. The ion source and transfer line temperatures were set, respectively, to 250 and 290 °C.

For selected ion monitoring (SIM) mode MS analysis, cells were scraped in 80% methanol and phase separation was achieved by centrifugation at 4 °C. The methanol-water phase containing polar metabolites was separated and dried using a vacuum concentrator. Dried polar metabolites were dissolved in 20 μL of 2% methoxyamine hydrochloride in pyridine (Pierce, Thermo Fisher Scientific) and held at 37 °C for 2 h. After dissolution and reaction, 80 μL *N*-*tert*-Butyldimethylsilyl-*N*-methyltrifluoroacetamide with 1% *tert*-Butyldimethylchlorosilane MBTSTFA + 1% TBDMCS (Thermo Fisher Scientific) was added and samples were incubated at 60 °C for 60 min. Gas chromatographic runs were performed with helium as carrier gas at 0.6 mL/min. The split inlet temperature was set to 250 °C and the injection volume of 1 μL. The GC oven temperature ramp was from 70 to 280 °C. The first temperature ramp was from 70 to 140 °C at 3 °C/min. The second temperature ramp was from 140 to 180 °C at 1 °C/min. Finally, the latest temperature ramp was from 180 to 280 °C at 3 °C/min. The data acquisition rate was 10 Hz. For the Quadrupole, an EI source (70 eV) was used. The ion source and transfer line temperatures were set, respectively, to 250 and 290 °C. For the determination of relative metabolite abundances, the integrated signal of all ions for each metabolite fragment was normalized by the signal from norvaline and the per cell number.

### 2.8. Metabolomics Data Analysis

Chromatograms and spectra were initially analyzed using MassHunter Qualitative Analysis and then processed using Profinder (Agilent Technologies, Cernusco sul Naviglio, Italy). Profinder returned 3624 aligned compounds using the following filters: *m*/*z* range (100–1000 *m*/*z*), absolute peak height (>10,000 counts), number of ions required (≥5 ions).

Data generated were exported as compound exchange format files (.CEF file) and subjected to Mass Profiler Professional (MPP, Agilent Technologies). Data were median log transformed and subjected to statistical analysis using Tukey’s honest significant difference (HSD)-corrected analysis of variance (ANOVA). Differentially expressed *m*/*z* entities were selected to have an adjusted *p*-value ≤ 0.05 in the ANOVA test, with a Benjamini and Hochberg correction. This analysis returned 1953 entities that were then further processed using MPP for the principal component (PCA) and for clustering analyses. Unsupervised hierarchical clustering was performed using the list of differentially expressed entities (i.e., metabolites) and conditions (resistance versus sensitivity) and Euclidean correlation and Ward’s linkage rule were used as measure of similarity. Metabolites identification was performed using MPP and Fihen Metabolomics retention time locking (RTL) library (Agilent G1676AA) as a reference. A Table with the identification output is provided as Supplementary Table S1.

### 2.9. Seahorse XFe96 Metabolic Assays

Cells were seeded in XFe96 cell culture plates with 1.5–2 × 10^4^ cells per well and subjected to the extracellular flux (XF) glycolytic rate assay and/or to the XF mito stress test. For the XF glycolytic rate assay, PDR cells were maintained in routine culture conditions while PDS cells were either left in basal medium or challenged for 72 h with 1 µM palbociclib to evaluate the effects of palbociclib acute treatment. After 3 days, media were replaced with XF assay medium supplemented with 1 mM pyruvate, 2 mM glutamine, 10 mM glucose and incubated at 37 °C in a non-CO_2_ incubator for 1 h before the analysis. The glycolytic rate assay discriminates between the extracellular acidification (ECAR) that is dependent on mitochondrial-derived CO_2_ and that of glycolysis by concomitantly measuring the amount of oxygen consumed (OCR) by the cells, hence calculating the total proton efflux rate (PER). This analysis is performed in real-time by measuring ECAR and OCR after Rotenone/Antimycin A (0.5 µM) and 2-DG (50 mM) administration. For the XF Mito Stress test, cells were cultured in a basal medium that was replaced 24 h post-seeding with XF base medium supplemented with 25 mM glucose, 2 mM glutamine and 1 mM sodium pyruvate. Cells were incubated for 1 h at 37 °C in a non-CO_2_ incubator before the analysis. Mito Stress test analysis reveals basal respiration, maximal respiration and the ability of the cells to exploit mitochondrial oxidative metabolism. This analysis is performed by real-time measurement of ECAR and OCR after a sequence of compounds that interfere with the electron transport chain: oligomycin (1 μM), carbonyl cyanide-4 (trifluoromethoxy) phenylhydrazone (FCCP) (1 μM) and Rotenone/Antimycin A (0.5 μM). Protein quantification was used to normalize the results of Mito stress and Glycolytic rate test.

### 2.10. Analysis of Human Datasets

Survival analysis was performed using Kaplan-Meier curve log-rank testing, using Cutoff finder [15] for best (202934_at) *HK2* high- and low-expression selection. The curated dataset of ER^+^ breast cancers was created using Km-plotter [16]. The relapse-free survival (RFS) data of patients belong to the following datasets: GSE45255, GSE37946, GSE2603, GSE21653, GSE20711, GSE19615, GSE17907, GSE16391, E-MTAB-365. The overall survival (OS) data of patients belong to the following datasets: GSE45255, GSE37946, GSE20711. *HK2* expression is from patient-derived material at diagnosis analyzed accordingly to the original studies. Information on normalization methods and multivariate analysis are available online at the KMplotter website and have been described in [16].

### 2.11. Statistical Analysis

Statistics were performed using Prism 8 (GraphPad Software, San Diego, CA, USA). Unless stated otherwise, all numerical data are expressed as the mean ± standard error of the mean (SEM). All experiments were conducted at least 3 times independently, with 3 or more technical replicates for each experimental condition tested. Unless stated otherwise, comparisons between 2 groups were made using the two-tailed, unpaired Student’s t-test. Comparisons between multiple groups were made using one-way ANOVA. Bonferroni and Dunnett post-testing analysis with a confidence interval of 95% was used for individual comparisons as reported in figure legends. Multivariate Cox analyses on the cohort of patients analyzed were generated using KM-plotter. Statistical significance was defined as: * *p* < 0.05; ** *p* < 0.01; *** *p* < 0.001, **** *p* < 0.0001; when differences were not statistically significant or the comparison not biologically relevant no indication were reported in the figures.

## 3. Results

### 3.1. Palbociclib Impacts on the Expression of Key Players Involved in Glucose Catabolism

To investigate the metabolic reprogramming occurring during response and at resistance to palbociclib, we first performed gene expression and protein analysis of key metabolic players involved in glucose metabolism on a panel of palbociclib sensitive (PDS) cells, in the presence or absence of 1 μM palbociclib, and PDR derivatives. The panel contains ER^+^ cell lines with differential HER2 status (i.e., ZR75-1 and T47D are ER^+^/HER2^−^, BT474 and MDA-MB-361 are ER^+^/HER2^+^) and has been previously characterized [13]. However, no common transcriptional programs were associated with palbociclib resistance, since cell-type specific features seem to dictate unsupervised hierarchical clustering based on the transcriptomic analysis as detailed in [13]. Since CDK4/6 inhibitors have been reported to perturb glucose dependent metabolism [17], we initially monitored in the isogenic cell lines’ established traits of cells undergoing aerobic glycolysis.

**Figure 1:**
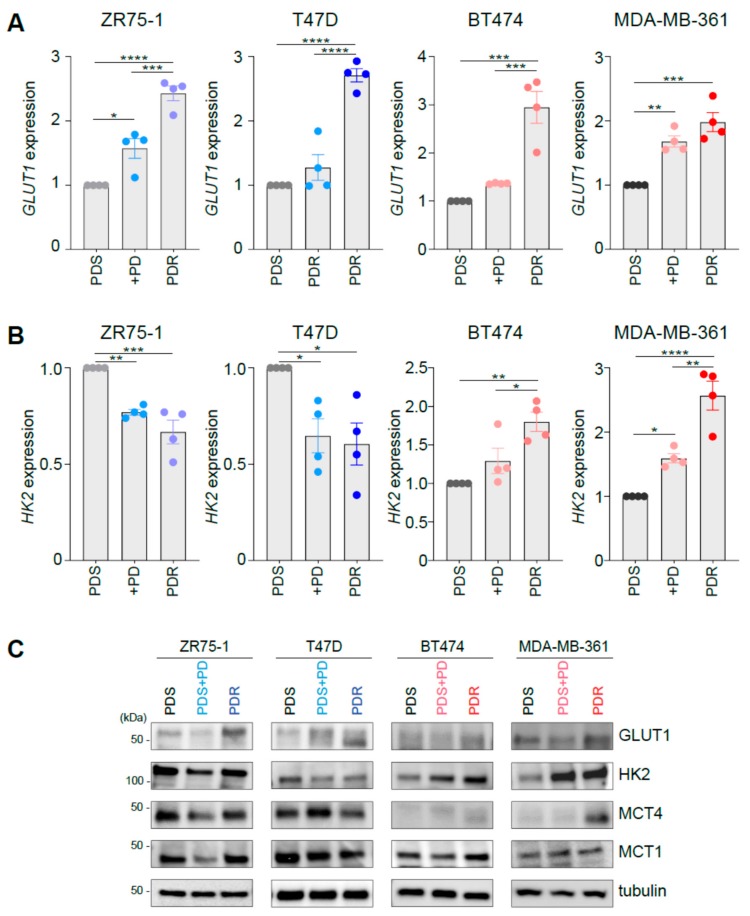
Palbociclib impacts on the expression of key players involved in glucose catabolism. (**A**, **B**) Palbociclib-resistant (PDR) and parental sensitive (PDS) cells, in presence or absence of 1µM palbociclib (PD), were subjected to quantitative real-time polymerase chain reaction (qRT-PCR) analysis using the assays described in the figure. Relative expression is shown using the PDS cells as comparator. Each dot represents a biological replicate. Gray shades are used for PDS cells, blue shades when PD is administrated to human epidermal growth factor receptor 2 (HER2^−^) cells (either PDS or PDR), red shades when PD is administrated to HER2^+^ cells (either PDS or PDR). Data represent means ± standard errors of the mean (SEMs). One-way analysis of variance (ANOVA); Bonferroni corrected; * *p* < 0.05; ** *p* < 0.01; *** *p* < 0.001; **** *p* < 0.0001. (**C**) Total protein lysates from PDS (either in the presence or absence of 1 µM palbociclib, PD) and PDR cells were subjected to Western blot analysis, as indicated.

qRT-PCR analysis revealed enhanced expression levels of *GLUT1* in all the PDR cells analyzed (Figure 1A). GLUT1 is found overexpressed and can contribute to enhanced glucose uptake in many tumor cells [18]. However, the expression of the rate-limiting enzyme of the glycolytic pathway hexokinase 2 (HK2) was differently regulated in PDR cells, depending on *HER2* amplification status. Indeed, HER2^−^ ZR75-1-PDR and T47D-PDR cells showed a modest but significant decrease in *HK2* expression (Figure 1B, left), whereas HER2^+^ PDR cell lines (i.e., BT474 and MDA-MB-361) increased its expression (Figure 1B, right) compared to their PDS counterpart. Interestingly, palbociclib administration to PDS cells induced a similar gene expression pattern for *HK2* and *GLUT1* (Figure 1A,B), suggesting that acute (3-day) treatment may promote a metabolic phenotype switch which favors therapy resistance. However, for the sake of completeness, palbociclib administration was shown to induce growth arrest and senescence in the sensitive cells [13] and therefore this response may be potentially linked to change in the cell proliferation rate. *HK2* expression changes observed in PDR cells and palbociclib-treated parental cells were confirmed at the protein level (Figure 1C). However, we also know that (i) different isoforms of HK may be expressed and therefore being involved in this rate-limiting reaction and (ii) HK2 expression does not directly measure HK enzymatic activity, hence the potential discrepancies observed at mRNA and protein expression levels between HK2 and GLUT1 render the identification of glycolysis dependency difficult and should be validated at the functional level. Since enhanced glucose uptake may be a prerogative of cells that experience aerobic glycolysis, we investigated the levels of monocarboxylates transporters MCT1 and MCT4 that, despite having different affinity for lactate (MCT1: Km ~3.5–10 mM, MCT4: Km ~22–28 mM) [19], can both function as lactate exporters in glycolytic cells, i.e., when lactate and pH gradient are driving lactate efflux [20]. Interestingly, western blot analysis showed enhanced levels of either MCT1 or MCT4 in ER^+^/HER2^+^ PDR cells when compared to the sensitive counterpart, whereas decreased levels of MCT1 or MCT4 were observed in the ER^+^/HER2^−^ PDR cells (Figure 1C and Supplementary Figure S1A). Palbociclib acute treatment induced a decrease of MCT1 or MCT4 levels in the ER^+^/HER2^−^ PDS cell lines and a small enhancement was observed in the ER^+^/HER2^+^ PDS cells (Figure 1C).

### 3.2. Resistant Cells Show Comparable Growth Rates When Compared to Sensitive Counterpart While Increasing Glucose Uptake

**Figure 2:**
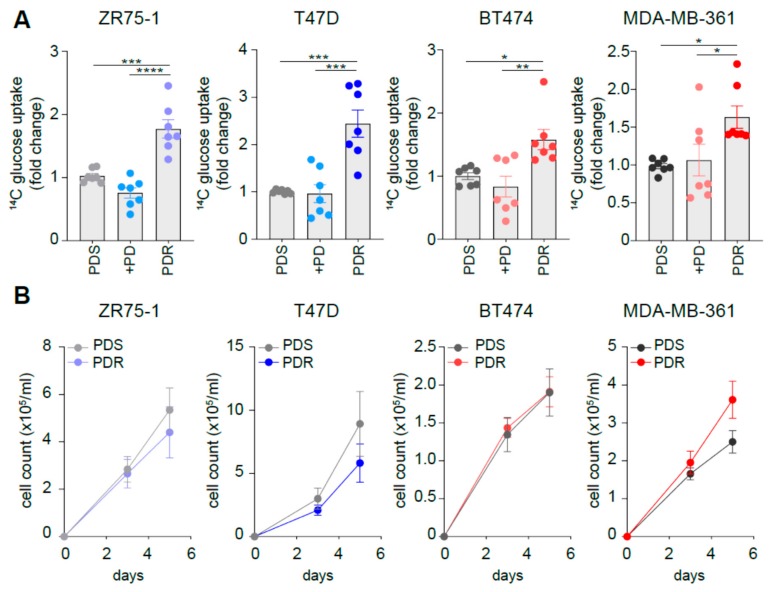
Resistant cells show comparable growth rates when compared to sensitive counterpart while increasing glucose uptake. (**A**) ^14^C-glucose uptake was measured in PDS (either in presence or absence of 1 µM palbociclib, PD) and PDR cells. The relative uptake capacity is shown using PDS cells as comparator. Each dot represents a biological replicate. Gray shades are used for PDS cells, blue shades when PD is administrated to HER2^−^ cells (either PDS or PDR), red shades when PD is administrated to HER2^+^ cells (either PDS or PDR). Data represent means ± SEMs. One-way ANOVA; Dunnett corrected; * *p* < 0.05; ** *p* < 0.01; *** *p* < 0.001; **** *p* < 0.0001. (**B**) No significant reduction in cell survival between PDS and PDR derived cells was observed by cell counting within the 5-day time range. Data represent means ± SEMs. Two-way ANOVA; Bonferroni corrected.

We then investigated whether the changes in the expression levels of the metabolic players could have a functional implication. Indeed, the increased *GLUT1* expression observed in the PDR cells is paralleled by enhanced glucose uptake, as monitored by tracing analysis using radiolabeled glucose (Figure 2A). However, despite 3-day palbociclib treatment enhanced *GLUT1* expression levels also in the sensitive cells, we did not observe enhanced glucose uptake in these conditions (Figure 2A). Importantly, the changes observed in metabolic genes expression and glucose uptake were not dependent on the survival of the sensitive and resistant cells, since no significant differences in growth rate were observed in a 5-day time-course experiment (Figure 2B and Supplementary Figure S1B), in line with a previous report [13]. To avoid any confounding effects of proliferation, all the metabolic-related analyses performed in the current study were performed between 24 and 72 h.

### 3.3. Human Epidermal Growth Factor Receptor 2 (HER2) Status Impacts on Glucose Catabolism and Reveals Different Glucose Dependency during Acute and Chronic Palbociclib Administration

To evaluate whether the enhanced glucose uptake of the PDR cells was paralleled by increased exploitation of glucose-dependent catabolism, we performed Seahorse analysis. In particular, glycolytic rate assay allows discriminating between the Extracellular Acidification Rate (ECAR) that is dependent on mitochondrial-derived CO_2_ and that of glycolysis, by concomitantly measuring the Oxygen Consumption Rate (OCR) by the cells, hence calculating the total Proton Efflux Rate (PER). Cells were subjected to real-time measurement of ECAR and OCR following mitochondrial function inhibition using Rotenone and Antimycin A (Rot/AA) followed by glycolysis inhibition using 2-deoxyglucose (2-DG).

**Figure 3:**
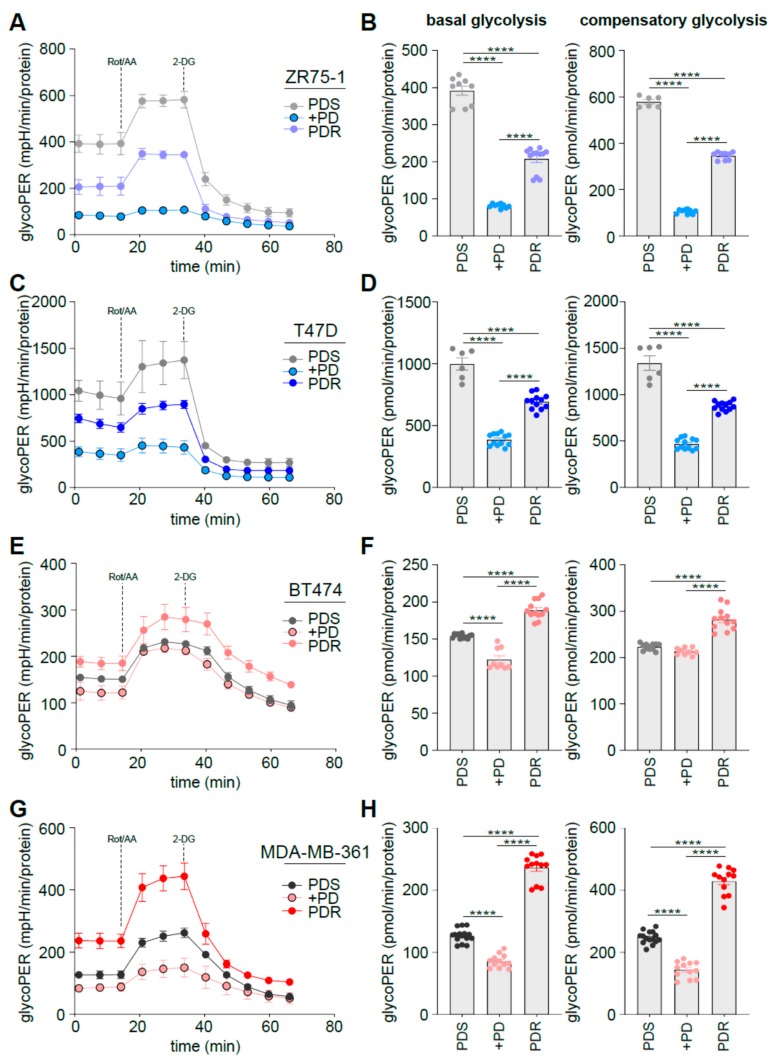
HER2 status impacts on glucose catabolism and reveals different glucose dependency during acute and chronic palbociclib administration. (**A**, **C**, **E**, **G**) PDS (either in presence or absence of 1 µM palbociclib, PD) and PDR cells were subjected to seahorse XFe96 glycolytic rate analysis and glycolytic proton efflux rate (glycoPER)) was measured in real time and normalized on protein levels. (**B**, **D**, **F**, **H**) Basal and compensatory (i.e., when electron transport chain is impaired) glycolytic capacity was calculated as described in Method Details, based on the extracellular acidification rate (ECAR) and oxygen consumption rate (OCR) after the administration of the respiratory complex I inhibitor rotenone, together with the respiratory complex III inhibitor antimycin A following by the glycolysis inhibitor 2-deoxy-glucose (2-DG). Data represent means ± SEMs. Each dot represents at least three technical replicates from three biological replicates. Blue dots are from HER2^−^ cells, red from HER2^+^. One-way ANOVA; Dunnett’s corrected; **** *p* < 0.0001.

ER^+^/HER2^−^ ZR75-1 and T47D PDR cells significantly decreased the overall glycoPER levels when compared to the PDS counterpart (Figure 3A,C). Accordingly, basal and compensatory glycolysis were significantly decreased (Figure 3B,D). Comparable data were obtained in the ER^+^/HER2^−^ MCF7 cells (Supplementary Figure S1C). Conversely, the ER^+^/HER2^+^ BT474 and MDA-MB-361 cells enhanced glycoPER levels (Figure 3E,G) and subsequently their basal and compensatory glycolysis (Figure 3F,H) when they become PDR. M oreover, palbociclib administration reduces basal and compensatory glycolysis in both HER2^−^ and HER2^+^ sensitive cells, suggesting that the metabolic behavior in response to palbociclib in the sensitive cells may be different from the metabolic phenotype of the resistant cells, at least in the ER^+^/HER2^+^ subset.

**Figure 4:**
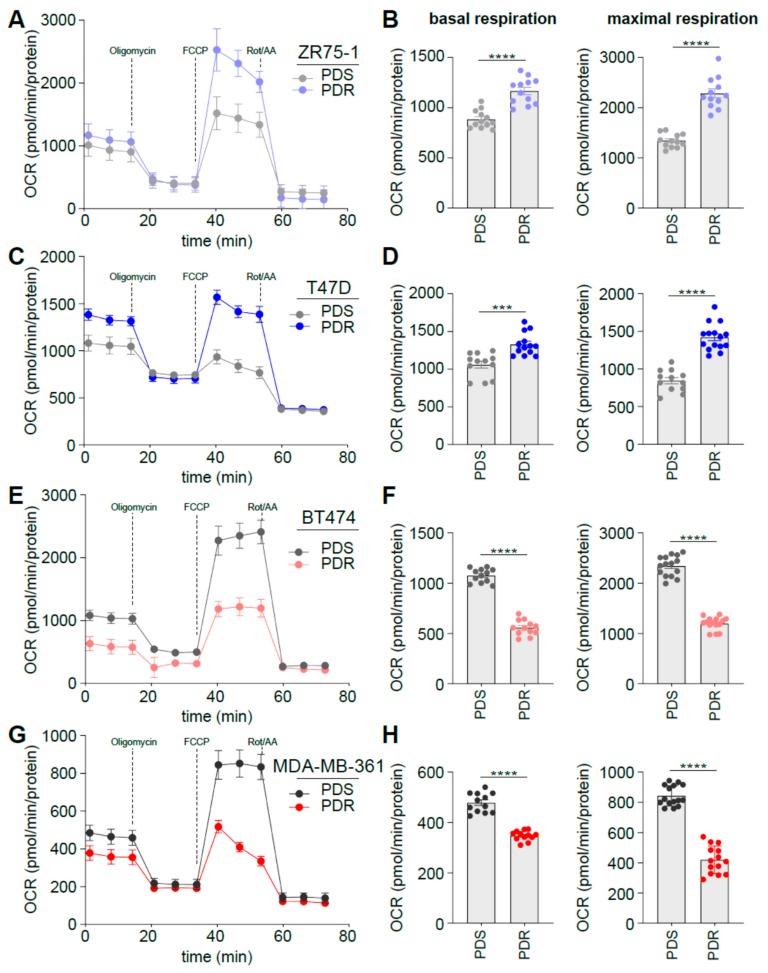
HER2 status impacts on glucose dependent oxidative phosphorylation in palbociclib resistance. (**A**, **C**, **E**, **G**) PDS and PDR cells were subjected to Seahorse XFe96 Mito Stress Test analysis and OCR was measured in real time and normalized on protein levels. (**B**, **D**, **F**, **H**) Basal and maximal respiration was calculated as described in Method Details, based on the OCR after the administration of the adenosine triphosphate ATP synthase inhibitor oligomycin, the proton uncoupler carbonilcyanide p-triflouromethoxyphenylhydrazone (FCCP), and the respiratory complex I inhibitor rotenone, together with the respiratory complex III inhibitor antimycin A. Each dot represents at least three technical replicates from three biological replicates. Data represent means ± SEMs. One-way ANOVA; Dunnett’s corrected; *** *p* < 0.001; **** *p* < 0.0001.

We have reported previously that during endocrine therapy resistance cells become more glycolytic while acquiring metabolic plasticity, a feature that renders metabolic targeting challenging [21,22]. Since aerobic glycolysis seems to play an important role in palbociclib resistance, we evaluated whether the PDR cells could exploit mitochondrial oxidative phosphorylation under metabolic stress conditions. OCR was measured in real-time following oligomycin, FCCP and Rotenone/Antimycin A, administrated at different time points to evaluate basal and maximal oxidative respiration. In line with the glycoPER data, HER2^−^ PDR ZR75-1 and T47D cells displayed enhanced levels of OCR and subsequent basal and maximal respiration when compared to the sensitive counterpart (Figure 4A–D). Conversely, HER2^+^ PDR BT474 and MDA-MB-361 cells showed reduced OCR, basal and maximal oxidative metabolism (Figure 4E–H), hence suggesting that the reduced respiration is counteracted by the enhanced aerobic glycolysis described by the glycolytic rate assay (Figure 3). Together these data highlight the existence of a metabolic reprogramming that is dependent on the HER2 status of the ER^+^ cell lines analyzed, suggesting potential therapeutic scenarios that may differ based on the expression of HER2 on the subset of the cells analyzed. These data are in line with previous studies showing that targeting glycolysis may have a synergistic effect in HER2^+^ breast cancer in combination with anti-HER2 therapy [23,24,25].

### 3.4. Targeting Glycolysis Resensitizes ER^+^/HER2^+^ Palbociclib-Resistant (PDR) Cells to Palbociclib

**Figure 5:**
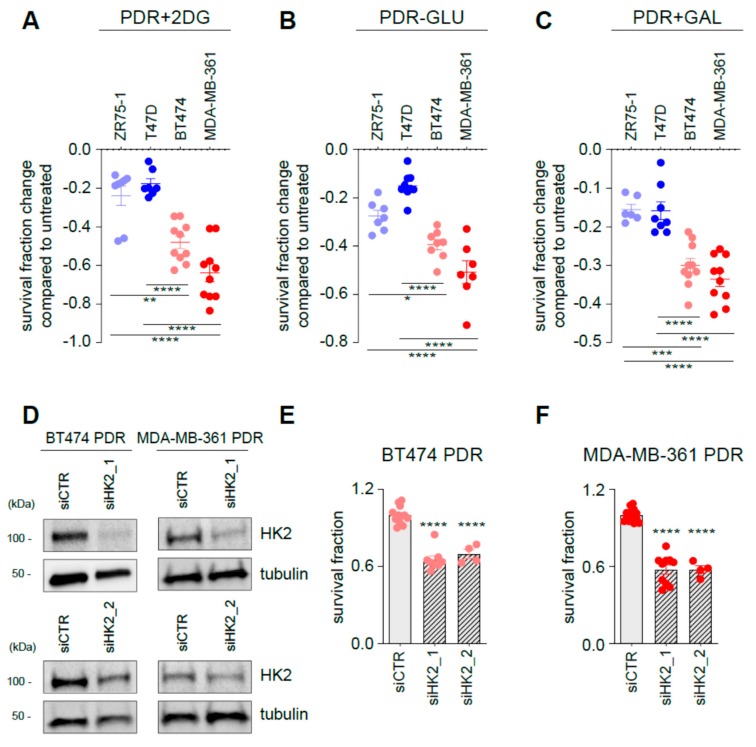
Targeting glycolysis resensitizes ER^+^/HER2^+^ PDR cells to palbociclib. PDR cells were either treated for 3 days in the presence of 2-DG (**A**), glucose-deprived medium (**B**) or in a medium in which glucose is replaced by galactose (**C**). Data are presented as fold change survival fraction of treated versus vehicle-treated or basal-cultured cells. Each dot represents at least three technical replicates from three biological replicate. Shades of blue dots are for HER2^−^ PDR cells, shades of red dots for HER2^+^ PDR cells. Data represent means ± SEMs and were compared to vehicle-treated or basal-cultured conditions using one-way ANOVA; Dunnett corrected; * *p* < 0.05; ** *p* < 0.01; *** *p* < 0.001; **** *p* < 0.0001. (**D**) Total protein lysates from ER^+^/HER2^+^ PDR cells transfected with the oligos as described in the figure for 72 h were subjected to Western blot analysis, as indicated. (**E**, **F**) Survival fraction changes were measured in cells transfected as indicated in the Figure. Each dot represents at least three technical replicates from three biological replicates. Data represent means ± SEMs and were compared to non-targeting control siRNA (siCTR)-treated cells using Student t test; **** *p* < 0.0001.

Since glucose catabolism seems to be a major contributor to palbociclib response and emergence of palbociclib resistance, we interfered with the glycolytic pathway with an array of different approaches. First, PDR cells were exposed to 2-DG, a compound that can be phosphorylated but is not further metabolized by cells. 2-DG treatment reduces of ~20% the survival fraction of HER2^−^ PDR cells when compared to control untreated PDR cells (Figure 5A and Supplementary Figure S1D, blue symbols). Importantly, the reduction after 2-DG treatment in the HER2^+^ PDR BT474 and MDA-MB-361 cells was 48% and 64% (Figure 5A, red symbols), respectively. Comparable results were obtained when cells were cultured in the absence of glucose (Figure 5B and Supplementary Figure S1D) and in a medium in which glucose is replaced by galactose (Figure 5C and Supplementary Figure S1D), a condition that increases reliance on oxidative phosphorylation for energy production [26]. To investigate whether the presence of palbociclib was necessary to induce the survival changes when glycolysis was targeted, the same approach was performed in PDR cells in the absence of palbociclib. Treatments were effective only when palbociclib was co-administrated to the cells (Supplementary Figure S2A). These results support the concept that glycolysis targeting resensitizes PDR cells to palbociclib treatment. To confirm the crucial role of glycolysis in palbociclib resistance of the HER2^+^ cells, we further targeted glycolysis by silencing HK2 in BT474 and MDA-MB-361 PDR cells (Figure 5D). Importantly, HK2 targeting reduces cell survival of both BT474 PDR (Figure 5E) and MDA-MB-361 PDR (Figure 5F), confirming the data generated by non-genetic interference.

### 3.5. Targeting Glycolysis Potentiates Palbociclib Effects on ER^+^/HER2^−^ Parental Cells

**Figure 6:**
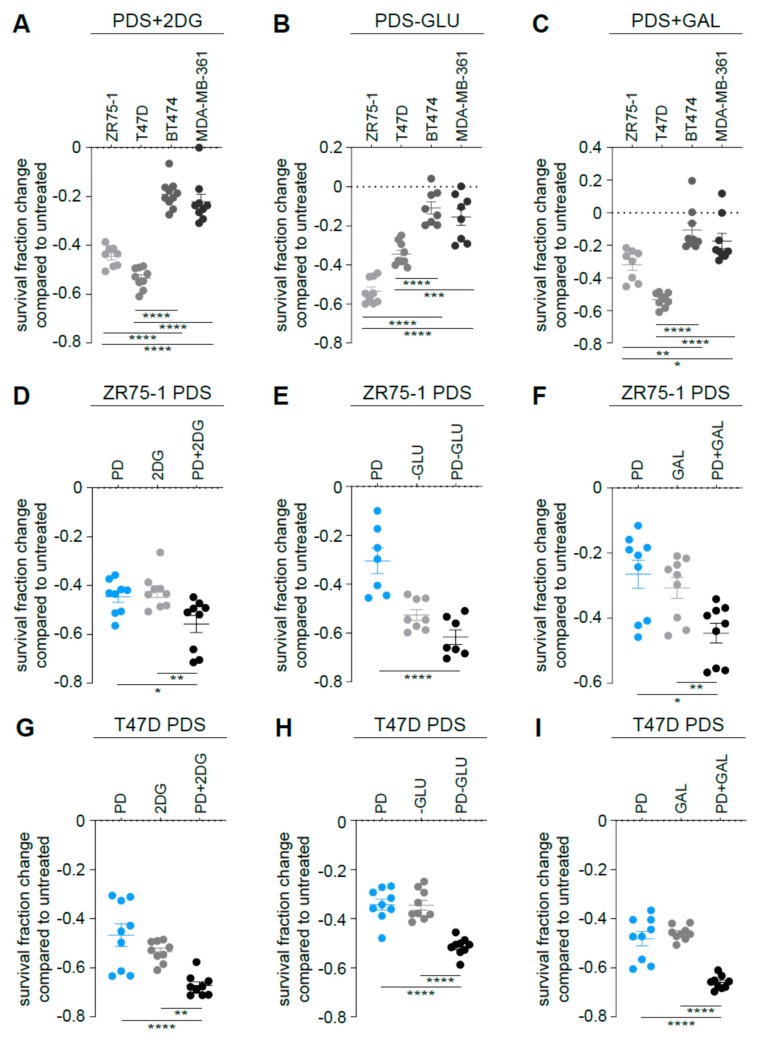
Targeting glycolysis potentiates palbociclib effects on ER^+^/HER2^−^ parental cells. PDS cells were either treated for 3 days in the presence of 2-DG (**A**), glucose-deprived medium (**B**) or in a medium in which glucose is replaced by galactose (**C**). Data are presented as fold change survival fraction of treated versus vehicle-treated or basal-cultured cells. Each dot represents at least three technical replicates from three biological replicates. Data represent means ± SEMs and were compared to vehicle-treated or basal-cultured conditions using one-way ANOVA; Dunnett corrected; * *p* < 0.05; ** *p* < 0.01; *** *p* < 0.001; **** *p* < 0.0001. ER^+^/HER2^−^ PDS cells were either treated for 3 days in the presence of 2-DG (**D**, **G**), glucose-deprived medium (**E**, **H**) or in a medium in which glucose is replaced by galactose (**F**, **I**) either in presence or absence of 1 µM palbociclib (PD). Data are presented as fold change of the survival fraction of treated versus vehicle-treated or basal-cultured cells. Each dot represents at least three technical replicates from three biological replicates. Gray shades are used for PDS cells, blue dots when PD is administrated to HER2- PDS cells. Data represent means ± SEMs and were compared to vehicle-treated or basal-cultured conditions using one-way ANOVA; Dunnett corrected; * *p* < 0.05; ** *p* < 0.01; **** *p* < 0.0001.

Since ER^+^/HER2^−^ PDS cells displayed high glycolytic dependency accordingly to the metabolic characterization (Figure 1, Figure 2 and Figure 3), we hypothesized that impairing glycolysis in the parental cells could impact the survival fraction of those cells and potentiate the effects of the palbociclib. Indeed, 2-DG administration (Figure 6A and Supplementary Figure S1D), glucose-deprived medium (Figure 6B and Supplementary Figure S1D) and galactose-containing medium (Figure 6C and Supplementary Figure S1D) had a higher impact on cell survival of ER^+^/HER2^−^ MCF7, ZR75-1 and T47D PDS cells when compared to ER^+^/HER2^+^ parental cells. Accordingly, we hypothesized that combining palbociclib with a glycolysis inhibitor may be more efficient than using palbociclib as monotherapy. Indeed, 2-DG administration (Figure 6D,G and Supplementary Figure S1D), glucose-deprived medium (Figure 6E,H and Supplementary Figure S1D) and galactose-containing medium (Figure 6F,I and Supplementary Figure S1D) enhanced the effect of palbociclib in inhibiting ER^+^/HER2^−^ cell survival. No impact was observed in the ER^+^/HER2^+^ PDS cell lines (Supplementary Figure S3).

### 3.6. Metabolomic Profiling Identifies a Distinct Intracellular Metabolites Pattern between HER2^+^ and HER2^−^ ER^+^ PDR Cells

The functional metabolic profile of the PDR cells suggests that the four isogenic cell lines are characterized by different metabolic behavior at the time of palbociclib resistance. These metabolic differences may result in a different intracellular metabolites composition. To address this hypothesis, we performed gas chromatography-mass spectrometry (GC–MS) analysis. Three independent biological replicates of PDS and PDR cell lines were subjected to cell lysis, metabolites extraction, and subsequent derivatization before processing to GC–MS. The chromatogram and the *m*/*z* entities of each sample were matched to the Fiehn library that allows, after Mass Profiler Professional software processing, the identification of ~1K metabolites [27]. However, it was not possible to identify all the *m*/*z* entities generated by GC–MS, although differentially expressed between different samples (Supplementary Table S1).

**Figure 7:**
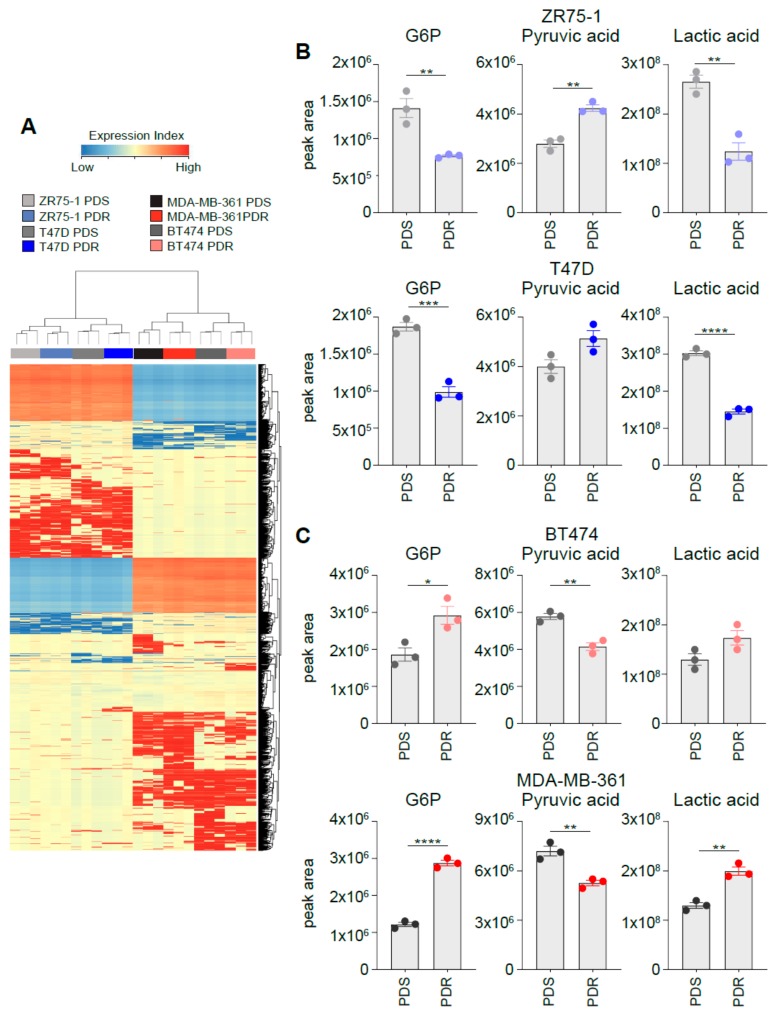
Metabolomic profiling identifies a distinct intracellular metabolites pattern between HER2^+^ and HER2^−^ ER^+^ PDR cells. (**A**) Unsupervised hierarchical clustering and heat map of the entities derived by the cell lysis, metabolites extraction, and subsequent derivatization before processing to gas chromatography–mass spectrometry (GC–MS) from three independent biological replicates of PDS and PDR cell lines. (**B**, **C**) Intracellular relative abundance of the following metabolites in ER^+^/HER2^−^ (B) and in ER^+^/HER2^+^ cells (C): glucose-6-phosphate, lactate and pyruvate. * *p* < 0.05; ** *p* < 0.01; *** *p* < 0.001; **** *p* < 0.0001.

Unsupervised hierarchical cluster analysis shows that the samples divide into HER2^−^ and HER2^+^ breast cancer cell lines and that, within these 2 groups, the samples cluster according to palbociclib sensitivity and resistance (Figure 7A). Importantly, Venn diagrams of the differentially regulated metabolites show little overlap between the different cell lines analyzed (Supplementary Figure S4 and Supplementary Table S1), reinforcing the concept that the genetic background (i.e., HER2 status) of each cell line has a strong influence on the metabolomic profile. Importantly, the differences observed in the glycolysis dependencies of the in vitro models described in the study could be recapitulated by monitoring the intracellular levels of lactate, pyruvate and glucose-6-phosphate, rate-limiting metabolites in sustaining the glycolytic flux. Metabolomic analysis performed using selected ion monitoring mode showed that the relative abundance of intracellular lactate is reduced in HER2^−^ T47D and ZR75-1 PDR cells (Figure 7B) and increased in HER2^+^ MDA-MB-361 and BT474 PDR cells when compared to parental PDS counterpart (Figure 7C). A comparable trend was observed in the levels of glucose-6-phospate (Figure 7B,C). Conversely, pyruvate levels were reduced in MDA-MB-361 and BT474 PDR cells and increased in T47D and ZR75-1 PDR cells when compared to PDS cells (Figure 7B,C). The reliance on potential exploitation of fermentation of the analyzed cells can be monitored by the lactate/pyruvate ratio (Supplementary Figure S5) that supports the functional metabolic analysis described in Figure 1, Figure 2, Figure 3, Figure 4, Figure 5 and Figure 6, highlighting enhanced glycolysis dependency in the MDA-MB-361 and BT474 PDR cells and reduced in the T47D and ZR75-1 PDR cells, when compared to the parental counterpart. Accordingly, TCA intermediates citrate, succinate, fumarate and malate were enhanced in the ER^+^/HER2^−^ PDR cells and decreased in the ER^+^/HER2^+^ PDR cells, when compared to the parental counterpart (Supplementary Figure S6).

### 3.7. Enhanced HK2 Expression Levels Identify a Subset of Poor Prognosis Tumors

**Figure 8:**
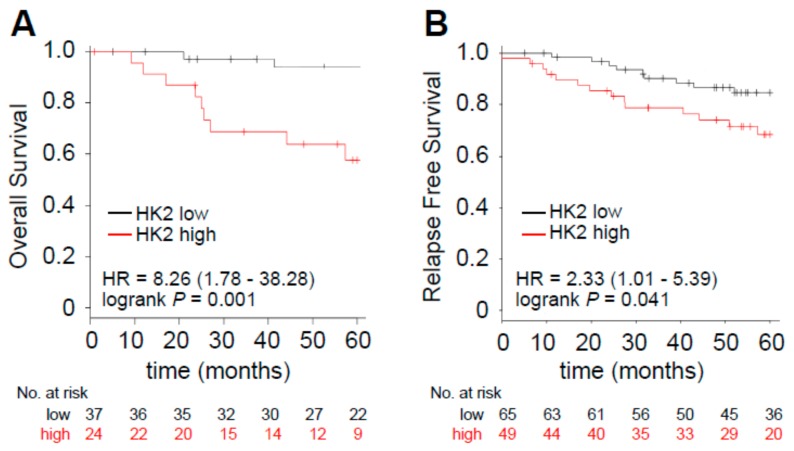
Enhanced *HK2* expression levels identify a subset of poor prognosis tumors. Kaplan–Meier analysis of overall survival (**A**) and relapse free survival (**B**) of a curated cohort of ER^+^/HER2^+^ patients divided into high- and low-expressing, as described in Method Details, for HK2 expression. The hazard ratio (HR), interval of confidence and log-rank Mantel–Cox *p* value are shown.

Since we could not retrieve a large dataset of patients that receive palbociclib and for which the clinical follow-up data and transcriptomic analysis are available, we investigated whether the glycolytic dependency is correlated to worse prognosis in the ER^+^/HER2^+^ subgroup which has been shown by the in vitro analysis to be dependent on glycolysis when resistance is acquired. Therefore, we explored the prognostic value of *HK2*, a key gene whose expression is an established hallmark of glucose catabolism [28]. We have already reported that breast cancers that express higher levels of *HK2* are characterized by worse prognosis in a cohort of ER^+^ breast cancer patients that received ET [21]. Here we investigated the prognostic value of *HK2* in a cohort of /ER^+^/HER2^+^ breast cancers and identified that higher expressing *HK2* tumors are characterized by reduced overall survival (OS, hazard ratio (HR) = 8.26, *p* = 0.001; Figure 8A) and relapse-free survival (RFS, HR = 2.33, *p* = 0.041; Figure 8B). Crucially, *HK2* prognostic value was independent of tumor proliferation as assessed by multivariate cox analysis using MKI67, ESR1 and HER2 expression levels (Table 1). These results suggest that *HK2* expression may be a further trait of aggressiveness in ER^+^/HER2^+^ tumors hence making those patients eligible for a more stringent follow-up or candidate to potential combinatorial therapy approaches.

## 4. Discussion

Metabolic reprogramming is an established trait of cancer cells and is implicated in therapy response and resistance [22]. In the current study we have used a previously characterized panel of isogenic ER^+^ breast cancer cell lines either sensitive or made resistant by chronical palbociclib exposure [13] and investigated their glucose metabolism. Although clinically CDK4/6 inhibitors are currently approved in combination with ET, the use of cells that are resistant to palbociclib (i.e., monotherapy) can generate biological data without the confounding effect of the additional agent used in the combinatorial treatment (i.e., ET or anti-HER2 therapy). This approach has been used previously and generated clinically relevant results, identifying cyclin E1 overexpression and Rb loss as genetic traits of palbociclib resistance [13,29,30]. Importantly, mammalian target of rapamycin complex 1 (mTORC1) activation is also reported to play an important role in the resistance mechanism and, currently, PI3K inhibitors (i.e., blocking PI3K/mTOR pathway) are in clinical trials in combination with CDK4/6 inhibitors [31]. Importantly, mTOR activation has also been reported to enhance glycolytic traits of the resistant cells, suggesting that glycolytic function may be under PI3K/mTOR signaling control in cancer and other pathologies [17].

**Figure 9:**
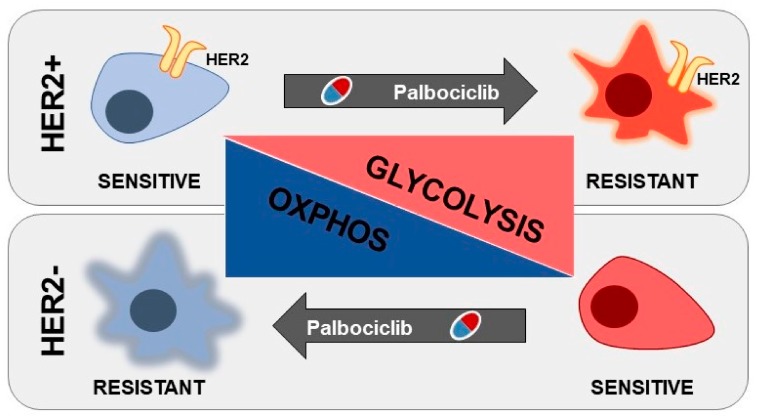
Graphical representation of the central carbon metabolic reprogramming during palbociclib resistance in ER^+^/HER2^+^ and ER^+^/HER2^−^ breast cancer cells.

The data herein presented highlight a glucose-dependency that is influenced by the genetic background of the cell lines used, i.e., HER2 amplification. In particular, ER^+^/HER2^−^ cell lines are characterized by high glucose exploitation when sensitive to palbociclib, and this dependency can be effectively targeted, hence potentiating the anti-proliferative effect of the drug. Conversely, ER^+^/HER2^+^ cells show a significant increase in glucose catabolic dependency when becoming resistant, and glycolysis targeting by multiple approaches is able to resensitize PDR cells to the initial therapy (Figure 9).

Often, in tumor cells, glucose is partially oxidized to pyruvate and subsequently fermented to lactate, even in the presence of oxygen [32,33]. This glucose metabolic exploitation is fundamental in tumorigenesis since it could provide both ATP and building blocks by fueling anabolic pathways that originate from glycolytic intermediates. It has been reported previously that the cyclin D1-CDK4 and cyclin D3-CDK6 pairs can regulate glucose metabolism [10,12] and that palbociclib administration reduces glucose metabolism in triple negative breast cancer in vitro models [34]. The rationale for investigating the glucose metabolic reprogramming associated with resistance to palbociclib is also supported by preclinical studies showing that (i) CDK4/6 inhibition enhances central carbon metabolism, the number of mitochondria and reactive oxygen species (ROS) production, a phenomenon associated with enhanced mTORC1 activity in pancreatic cancer models [17] and that (ii) CDK4/6 inhibitors efficacy is potentiated by autophagy inhibition in Rb-positive solid tumors [9]. Although the initial (basal) characterization of glucose uptake and *GLUT1* expression levels suggested a common mechanism of resistance since all the cell lines showed enhanced glucose avidity, independently of HER2 status, further functional metabolic analysis revealed a different metabolic phenotype. Indeed, Seahorse analyses showed that glycolysis dependency was reduced in ER^+^/HER2^−^ PDR cells while significantly increased in the ER^+^/HER2^+^ PDR cell lines. Accordingly, the exploitation of oxidative phosphorylation is inversely correlated with the glycolytic dependency, as monitored by oxygen consumption under basal and mitochondrial stress conditions. The discrepancies observed between glucose uptake and glycolysis dependency may be due to the fact that the enhanced glucose uptake observed in the ER^+^/HER2^−^ PDR cells does not result in increasing glucose catabolism but rather fuels alternative (i.e., anabolic) pathways that diverge from glycolytic intermediates such as pentose phosphate, hexosamine and/or serine biosynthesis. Indeed, palbociclib treatment in MCF7 has been shown to enhance pentoses levels, a readout of the oxidative branch of the pentose phosphate pathway [35]. These anabolic pathways would provide building blocks necessary for proliferating cells and nicotinamide adenine dinucleotide phosphate (NADPH) essential to counteract the potential oxidative stress enhancement that occurs in cells under therapeutic pressure.

The metabolic differences described have been exploited by targeting the priming step of glycolysis (i.e., HK2) and glycolytic dependencies in different contexts thus suggesting that targeting glucose metabolism may be more effective in the sensitive setting in the ER^+^/HER2^−^ subset while it can re-sensitize ER^+^/HER2^+^ PDR breast cancers to the initial therapy. Importantly, the differences described could also point to investigate the consequence of targeting the oxidative metabolism (e.g., targeting complexes in the electron transport chain) in the ER^+^/HER2^−^ resistant clinical scenario, more likely to occur in the near future, since palbociclib is currently used in the ER^+^/HER2^−^ cohort of patients.

Although ER^+^/HER2^+^ breast cancers are not currently eligible for CDK4/6 inhibitor therapy [36], preclinical data have shown that cyclin D1/CDK4/6/pRB axis may be deregulated and represent a key mechanism driving resistance to anti-HER2 therapies [37]. Importantly, breast cancer cells that survive lapatinib treatment show enhanced activation of cyclin D1/CDK4 complex, hence suggesting the importance of this pair for anti-HER2 response and subsequent relapse. A series of clinical trials are now enrolling ER^+^/HER2^+^ patients to test the efficacy of approaches combining palbociclib and anti-HER2 therapies in breast cancer (i.e., ClinicalTrials.gov identifier: NCT02448420-PATRICIA, NCT02530424-NA-PHER2 and NCT03644186-TOUCH) either as doublets or more complex combinations with the addition of endocrine therapies.

The metabolic analysis herein described reveals that enhanced glycolysis dependency might be a trait of ER^+^/HER2^+^ tumors that are prone to acquire resistance to palbociclib. Importantly, this propensity is revealed by monitoring HK2 expression levels, a trait that has been previously reported to be intimately interconnected with aerobic glycolysis. Kaplan–Meier analyses of overall and relapse-free survival in ER^+^/HER2^+^ breast cancer patients revealed that HK2 high-expressing tumors are characterized by worse prognosis, independently of HER2, ESR1 and proliferation status measured using MKI67 levels. However, these analyses do not directly prove the clinical significance of HK2 as a predictive marker in palbociclib treated ER^+^ patients and should be further validated in matched tissue biopsies obtained at diagnosis and at the time of disease progression on palbociclib treatment. Moreover, preclinical studies should reveal whether favoring glycolysis in the sensitive setting is sufficient to desensitize cells to palbociclib, hence favoring resistance.

In conclusion, the current study demonstrates a role for glucose metabolism in sustaining resistance to palbociclib. The implications of these results are multiple: (i) targeting glucose dependency is effective in preclinical in vitro models and warrants further investigations; (ii) since glucose metabolism can be monitored in patients using ^18^F-fluorodeoxyglucose (^18^F-FDG) PET/CT, understanding the correlation between ^18^F-FDG PET and palbociclib response could be a non-invasive methods to better tailor personalized therapies and monitor therapy response; (iii) the data that emerged in the HER2^+^ subset may help supporting biomarker discovery and potential treatment vulnerabilities in ER^+^/HER2^+^ cancers treated with CDK4/6 inhibitors. The in vitro data herein described needs to be further confirmed in vivo to validate whether tumor metabolic features may have a potential clinical value in palbociclib resistance either to follow up patients during their treatment or to develop new therapeutic strategies in combination with CDK4/6 inhibitors.

## Figures and Tables

**Table 1:** Multivariate Cox regression analysis (overall survival (OS), A) and (relapse-free survival (RFS), B).

	Parameters	*p* Value	Hazard Ratio (HR)
***A***	*MKI67*	0.909	0.93 (0.28–3.12)
	*ESR1*	0.118	0.11 (0.01–1.75)
	*HER2 (ERBB2)*	0.340	0.55 (0.16–1.88)
	*HK2*	0.0082	16.67 (2.07–134.21)
***B***	*MKI67*	0.739	0.87 (0.37–2.03)
	*ESR1*	0.0192	0.15 (0.03–0.73)
	*HER2 (ERBB2)*	0.3505	0.66 (0.27–1.59)
	*HK2*	0.0188	2.99 (1.2–7.47)

## Supplementary Materials

The following are available online at https://www.mdpi.com/2073-4409/9/3/668/s1: Figure S1: ER^+^/HER2^−^ MCF7 cells recapitulate the metabolic behaviour of the ER^+^/HER2^−^ breast cancer cells; Figure S2: PDR cells are re-sensitised to palbociclib when glycolysis is targeted; Figure S3: Targeting glycolysis alone or in combination with PD in ER^+^/HER2^+^ PDS cells has no major impact when compared to palbociclib alone; Figure S4: Commonly deregulated metabolites between the different PDR cell lines; Figure S5: Aerobic glycolysis is enhanced in ER^+^/HER2^+^ and reduced in ER^+^/HER2^−^ PDR cells when compared to PDS cells; Figure S6: TCA intermediates are enhanced in ER^+^/HER2^−^ and reduced in ER^+^/HER2^+^ PDR cells when compared to PDS cells; Table S1: Differentially expressed *m*/*z* entities between the different PDS and PDR cell lines.
